# Effect of Co-Existing Ions on Salinity Gradient Power Generation by Reverse Electrodialysis Using Different Ion Exchange Membrane Pairs

**DOI:** 10.3390/membranes12121240

**Published:** 2022-12-07

**Authors:** Tuğçe Zeynep Kaya, Esra Altıok, Enver Güler, Nalan Kabay

**Affiliations:** 1 Department of Chemical Engineering, Faculty of Engineering, Ege University, 35100 İzmir, Turkey; 2 Department of Chemical Engineering, Faculty of Engineering, Atilim University, 06830 Ankara, Turkey

**Keywords:** blue energy, co-existing ions, divalent ions, ion-exchange membranes, monovalent ions, reverse electrodialysis

## Abstract

This study investigates the influence of co-existing ions on the salinity gradient power generation performance of the reverse electrodialysis (RED) using three different commercial ion exchange membrane pairs. The feed solutions, including the mixture of two different salts, were prepared with 90 wt.% of NaCl and 10 wt.% of LiCl, KCl, CaCl_2_, MgCl_2_ or Na_2_SO_4_ by keeping the salt ratio between high concentrate solution and low concentrate solution constant as 1:30 (g/g) at various flow velocities (50, 125 and 200 mL/min). It was observed that the divalent ions exhibited a negative impact on the performance of the RED system due to their high valence and low ionic mobility depending on their high hydrated radius and low diffusion coefficients compared to those of the monovalent ions. On the other hand, the effect of the monovalent ions differed according to the properties of ion exchange membranes used in the RED stack. When the power generation performances of ion exchange membrane pairs employed in the RED stack were compared, it was considered that Neosepta AMX and CMX membranes provided the highest power density due to their low membrane thicknesses, low electrical resistances, and relatively high ion exchange capacities compared to other two commercial ion exchange membrane pairs.

## 1. Introduction

Due to the development of technological advancement, traditional energy sources are being consumed rapidly. The major energy sources such as fossil fuels or coal used worldwide caused some negative effects on nature. To reduce the probability of the energy sources depletion, the need for renewable energy sources should be eliminated, and the replenishable energy sources should be explored. The published reports and protocols about climate change represent to increase in the necessity of clean energy sources for reducing the negative impacts of conventional energy sources [1].

The non-hazardous and renewable energy sources are called renewable energy sources, and they include lots of energy sources that can be naturally renewable, such as solar, tidal, wind, geothermal, hydrodynamic, biomass, etc. [2,3,4,5]. On the other hand, these renewable sources also have some important disadvantages in their usage in power generation. One of these disadvantages is that their utilization can be influenced by climatic conditions. Due to changes in climatic conditions, some drawbacks to obtaining power can occur. This problem can be solved by storing the excess power to reduce the waste of energy by using an energy storage system (ESS), but this system requires an extra cost of operation and maintenance [6].

At this point, the energy production from the salinity gradient, which was explored by Pattle in 1954s, gained much more importance [7]. The main advantage of salinity gradient power generation is that this energy source is not affected by climatic conditions. The energy produced by the membrane systems depends on the difference in salinity gradient between two different salt solutions (seawater and river water) [8]. It is assumed that the energy produced from the salinity gradient may be reached 30 TW in World’s River mouths [9]. A study performed by Logan et al. [10] showed that the salinity gradient power generation could reach 2 TW by releasing the rivers into the sea. Additionally, when the purified wastewater flows into the sea, the power generation from the salinity gradient could be as high as 18 GW [8]. The driving force for the permeation of water is a difference in free energy between seawater and river water [10].

**Figure 1:**
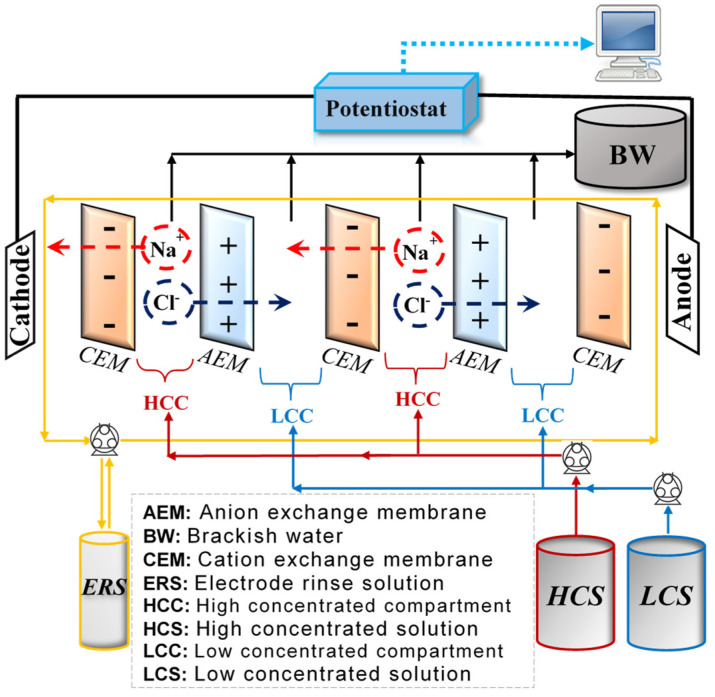
Detailed representation of the Reverse Electrodialysis (RED) process [14].

The RED method, which is the reversal process of electrodialysis (ED), is another membrane-based technology used in energy production. ED is a membrane-based method for separation, in which ions are pushed across an ion exchange membrane (IEMs) under the influence of an electric field, mostly used in the purification process [11]. The difference in chemical potential resulting from different salinities is used as a driving force in energy generation by the RED method. The RED stack consisted of alternately arranged cation exchange membranes (CEMs) and anion exchange membranes (AEMs) stacked between two electrodes (anode and cathode), occurring redox reactions (reduction in cathode and oxidation in anode) [12]. As shown in Figure 1, the alternating series of AEMs and CEMs provide an ion exchange process between high-concentrated solution (HCS) and low-concentrated solution (LCS). Additionally, an extra CEM is added facing to the cathode for the prevention of electrode abrasion due to the ionic interaction [13].

The salinity gradient results in a potential difference over each membrane, the so-called membrane potential. The overall potential difference of the RED stack is the sum of the potential differences over each membrane. The potential chemical difference causes the transport of ions through the IEMs from the HCS to the LCS. For example, when a sodium chloride solution is considered, the sodium ions pass through CEMs toward the cathode, and chloride ions move through the AEMs toward the anode. The oxidation reactions took place at the anode, while the reduction reactions took place at the cathode to reach electroneutrality. As a result, the ionic current is converted to the electrical current via redox reactions in electrodes. This electrical current and the potential difference over the electrodes result in power generation, and this can be measured by connecting a potentiostat to the RED module [10]. As a redox pair, K_4_Fe(CN)_6_ and K_3_Fe(CN)_6_ are generally used since they do not cause any power loss [15]. Guo et al. [16] used two homogeneous membranes (Yadeshi and Fujifilm membranes) to study the effect of co-existing ions. It was considered that the addition of co-existing ions caused to increase in the internal resistance of IEMs, and higher power density values were obtained by Fujifilm Type I membranes, having low internal resistance.

In real applications, another important topic is the ionic content in the feed solutions such as seawater and river water. The natural water sources contain various ions such as Na^+^, Cl^−^, K^+^, Li^+^, Mg^2+^, Ca^2+^, and SO_4_^2−^, and their characteristic properties, such as diffusion coefficient and hydrated radius of ions, are given in Table 1 [17,18,19]. It was reported that when seawater and river water, having 10% of the divalent salts, were used as feed solutions instead of feed solutions containing only NaCl, the cell potential was reduced by nearly 7% [18].

The membrane resistances were experimentally calculated using different IEMs for observing the RED performance in the presence of multivalent ions (Mg^2+^ and SO_4_^2−^) by Pintossi et al. [23]. Higher membrane resistances were observed for each IEM in the presence of MgCl_2_ and Na_2_SO_4_. In the same research, the current (I)-voltage (V) curves obtained were examined, and the negative effect of divalent ions on voltage was observed clearly. The influence of Mg^2+^ ions on the I-V curves was greater than that of SO_4_^2−^ ions due to the less permselectivity losses and electrical resistance raises [23].

The RED studies in the presence of monovalent ions (Li^+^, K^+^) are relatively less than those of multivalent ions. Guo et al. [16] studied the effect of co-existing ions such as K^+^, Mg^2+^, Ca^2+^, and SO_4_^2−^, with different IEMs (Fujifilm and Yadeshi membranes) at different temperatures (40 °C, 25 °C, and 10 °C). It was obtained that the increase in temperature resulted in an increase in the RED performance. The Fujifilm membranes gave higher power generation than the Yadeshi membranes due to the higher internal resistance of Yadeshi membranes. In addition, the negative effect of multivalent ions was clearly seen in the power density obtained. The effect of K^+^ ions was explained by comparing the activity coefficients of Na^+^ and K^+^ ions at different concentrations. It was stated that the differences in activity coefficients of Na^+^ and K^+^ ions increase with an increase in solution concentration. When the solution concentration is high (such as 2.0 mol/L), the activity coefficient of K^+^ ions (0.576) is lower than that of Na^+^ ions (0.670). On the other hand, the power density value obtained in the presence of K^+^ ions (~0.24 W/m^2^) was close to the power density value obtained with the feed solution having only Na^+^ ions (0.25 W/m^2^) at 25 °C [16].

Hong et al. [24] performed the modeling of power generation by implementing salinity conditions of the natural sources. They reported that the addition of MgSO_4_, Na_2_SO_4_, and MgCl_2_ to the feed solutions caused a decreasing effect on power generation by 9–20%.

In electro membrane studies, synthetic solutions prepared in the laboratory are used in studies to observe the operating performance of the system, and mostly single-component solutions are chosen for the studies to avoid possible interferences. However, in applications where real–natural-feed solutions are used, the solutions contain many different ions. This study is aimed to observe the changes in system performance by using species that can potentially be found in natural solutions to be used in the RED system. In addition, a more specific aspect of the work is that the typical salt ratio between river water and seawater (1:30) is preserved in RED tests by using specific concentrations of salt pairs. When the literature was examined, it was seen that RED studies were performed with feed solutions containing only NaCl salt. Additionally, co-existing ion effects on the RED system were generally examined using the same type of ion exchange membranes in the literature. Unlike the literature, power generation performances of the RED system were compared using homogeneous and heterogeneous ion exchange membranes in order to examine the effect of co-existing ions. Basically, the effect of both co-existing ions and different ion exchange membranes on the power generation performance of the RED system was analyzed in this study. The influence of the co-existing ions on the RED system performance was investigated by the use of commercial heterogeneous and homogeneous ion exchange membrane pairs (heterogeneous: Ralex AMH-PES and Ralex CMH-PES; homogeneous: Neosepta AMX and Neosepta CMX; Fujifilm AEM Type 2 and Fujifilm CEM Type 2). In order to keep the salt ratio constant at 1:30 (g/g) in the feed solutions, 10 wt.% of monovalent (Li^+^ and K^+^) and divalent (Mg^2+^, Ca^2+^, and SO_4_^2−^) ions from their salts (LiCl, KCl, CaCl_2_, MgCl_2_ or Na_2_SO_4_) were mixed with 90 wt.% of NaCl.

## 2. Materials and Methods

### 2.1. RED Tests

**Figure 2:**
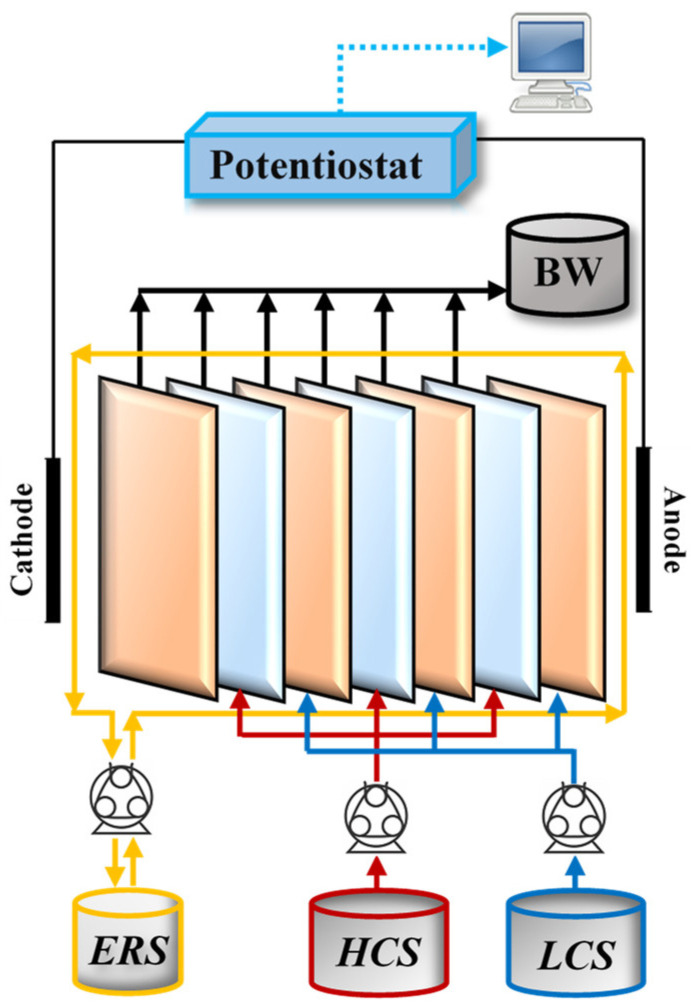
Flow diagram of the RED set-up [14].

The laboratory scale RED system was used in RED studies (STT Products B.V., Tolbert, Holland). The RED system includes a RED membrane stack, two feed solution tanks of 25 L for low-concentrated (LCS) and high-concentrated (HCS) salt solutions, and a tank of 2 L for electrode solution. The RED stack consists of inert spacers and silicone gaskets between the respective CEMs and AEMs. Two titanium electrodes coated with Ru/Ir are placed at two sides of the RED stack. Feed and electrode solutions were pumped with double and one-headed peristaltic pumps (Masterflex, Gelsenkirchen, Germany), respectively. Salt ratios of the feed solutions were adjusted to 1:30 (g/g) and measured by WTW 3110 model conductometer (Hach, Germany). Electrochemical measurements were carried out by Gamry Reference 3000 model potentiostat (Warminster, PA, USA). The flow diagram of the RED setup is shown in Figure 2.

All studies were repeated at least three times at each flow rate to obtain more accurate and precise results. The blank tests and calculations were performed like in our previous study [12]. Operating conditions are summarized in Table 2.

### 2.2. Ion Exchange Membranes (IEMs)

**Figure 3:**
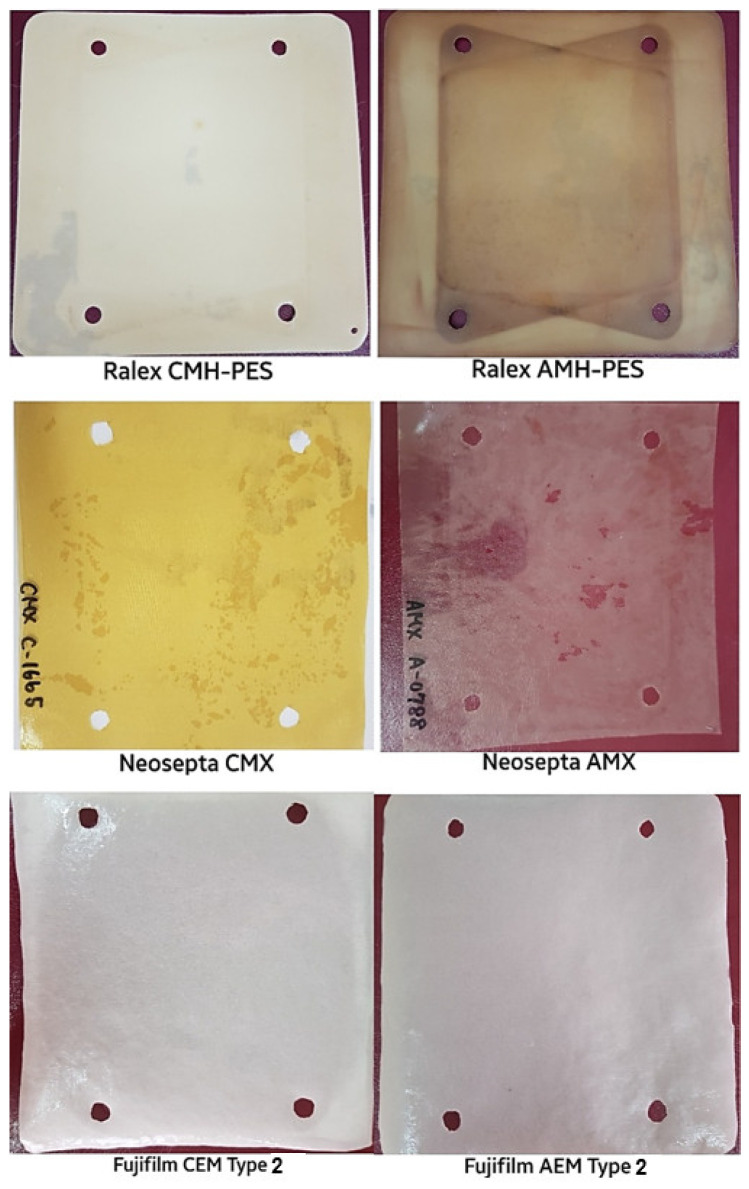
Images of commercial ion exchange membranes [14].

Different IEMs were used to investigate the influences of co-existing ions on power generation by the RED system. These commercial IEMs were either heterogeneous (Ralex AMH-PES and CMH-PES, (MEGA, Prague, Czech Republic)) or homogeneous (Neosepta AMX and Neosepta CMX (ASTOM Co., Tokyo, Japan); Fujifilm AEM Type 2 and Fujifilm CEM Type 2 (FUJIFILM Manufacturing Europe B.V., Tilburg, The Netherlands)). The membrane properties and images of each IEMs are shown in Table 3 and Figure 3, respectively. During RED tests, five membrane pairs were employed. By considering the membrane properties, Ralex membranes have heterogeneous structures, provide high mechanical strength, and have a non-uniform distribution of charges. Neosepta and Fujifilm membranes are homogeneous membranes which have uniform charge distribution. As shown in Table 3, the thinnest ion exchange membranes are Neosepta CMX and AMX membranes, while the thickest membranes are Ralex AMH-PES and CMH-PES membranes. The increase in membrane thickness caused to increase in the electrical resistance of IEMs. Therefore, Ralex CMH-PES and AMH-PES membranes have the highest electrical resistances compared to others. On the other hand, Ralex membranes (CMH-PES and AMH-PES) have high ion exchange capacities but also a reasonable swelling degree. The high electrical resistances of these membranes could be due to the relatively low charge densities of these membranes. According to the literature, if the IEMs have low membrane thickness, low electrical resistance, and high ion exchange capacity, their performance of power generation by the RED system could be highly enhanced [14,15,25].

### 2.3. Feed Solutions

For investigating the effects of co-existing ions on power generation by RED, various salts containing monovalent and divalent ions were used. The co-existing ions were monovalent ions (Na^+^, Li^+^, K^+^, and Cl^−^) and divalent ions (Mg^2+^, Ca^2+^ and SO_4_^2−^). The feed solutions were prepared as a mixture of 90 wt.% of NaCl and 10 wt.% of another salt containing monovalent or multivalent ions. In Table 4, the ingredients of high-concentrated and low-concentrated feed solutions are described with their concentrations.

### 2.4. RED Tests Performed for Investigation of Co-Existing Ion Effects

The experimental conditions of RED tests performed for the determination of the effect of different ions, such as Li^+^, K^+^, Ca^2+^, Mg^2+^, and SO_4_^2−^ were summarized in Table 5. High-concentrated (HC) and low-concentrated (LC) feed solutions were supplied to a RED stack having 5 membrane pairs at three different flow rates (50, 125, 200 mL/min). The flow rate of the electrode solution was adjusted to 300 mL/min in all RED tests. Each test was repeated three times to increase the accuracy and precision of the experimental results. The power density and open circuit voltage (OCV) values of RED tests were calculated by taking the average of the experimental results of each repetition.

### 2.5. Performance Analysis of the RED System

The performance of power generation in the RED system was analyzed by GAMRY Reference 3000 Model potentiostat-galvanostat analyzer. The electrochemical measurements obtained from the RED stack were transmitted to the computer by this analyzer. These measurements were chronopotentiometrically taken for 30 s at each flow rate value. The multistep chronopotentiometric method was used for the open circuit voltage (OCV, V) and current-voltage analysis. The OCV is the highest voltage value which is measured at zero current point. The power density (*P*) obtained in the current density range studied was found with related equations.

(1) W=V×I

(2) $$P=\frac{W}{2A_{m}N}$$

(3) $$i=\frac{I}{A_{e}}$$

The power (Watt) produced is calculated by multiplying each current (*I*) value by the potential difference (*V*) corresponding to each current value using Equation (1). Equation (2) defines the power density as the power produced per active membrane area (*A_m_*), where *P* is the power density (W/m^2^), *W* is the electrical power (*W*), A is the active membrane area (m^2^), and *N* is the number of membranes. Equation (3) is helpful to calculate the current density as the current per active electrode area (*A_e_*), where *I* is the current (*A*), and *i* is the current density (A/m^2^).

## 3. Results and Discussion

### 3.1. RED Studies Performed with Ralex Membranes

The commercial and heterogeneous anion exchange Ralex AMH-PES and cation exchange Ralex CMH-PES membranes were used for investigating the effects of co-existing ions on the power generation by RED.

**Figure 4:**
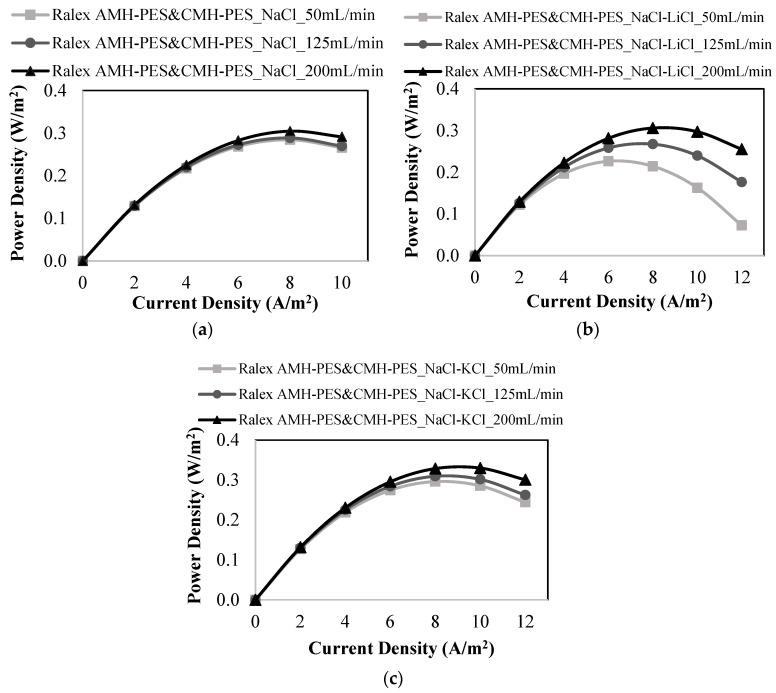
Power density vs. current density plots at different feed flow rates for the studies performed with (**a**) only NaCl, (**b**) a mixture of NaCl and LiCl, (**c**) a mixture of NaCl and KCl using Ralex AMH-PES & CMH-PES membranes.

As shown in Figure 4, the effect of monovalent ions was examined, and the maximum power density values in the presence of monovalent ions were obtained at the highest feed flow rate of 200 mL/min. As seen in Figure 4a, the study performed with feed solutions having only NaCl salt resulted in a power density of 0.305 W/m^2^ at 200 mL/min. When LiCl and NaCl salts were mixed in feed solutions, the maximum power density was obtained as 0.306 W/m^2^ (Figure 4b), which is very close to the power density result of the study performed with only NaCl (0.305 W/m^2^). It was seen in Figure 4c that the maximum power density increased to 0.330 W/m^2^ with the addition of KCl salt to the feed solutions.

The maximum power density results of the experiments carried out in the presence of monovalent ions (Na^+^, Li^+^, K^+^, and Cl^−^) were examined by comparing the hydrated radii and diffusion coefficients of Na^+^, Li^+^, and K^+^ ions. The power generation performance of the RED system was enhanced by the addition of Li^+^ and K^+^ ions to the feed solutions. As shown in Table 1, the increasing order of hydrated radius of monovalent cations is K^+^ < Na^+^ < Li^+^, while the increasing order of diffusion coefficient of monovalent cations follows Li^+^ < Na^+^ < K^+^. The hydrated radius and diffusion coefficient of the K^+^ ion is 3.31 × 10^−10^ m and 1.957 × 10^−9^ m^2^/s, respectively. The K^+^ ion has the highest ionic mobility due to the lowest hydrated radius and the highest diffusion coefficient values among the monovalent cations. Therefore, the transport of K^+^ ions through the cation exchange membranes is faster and easier than that of other cations. Thus, the highest power density (0.330 W/m^2^) was achieved in the presence of K^+^ ions in the feed solutions. On the other hand, the maximum power density obtained in the presence of Li^+^ ions was found as close to the power density of the study performed with only NaCl salt.

**Figure 5:**
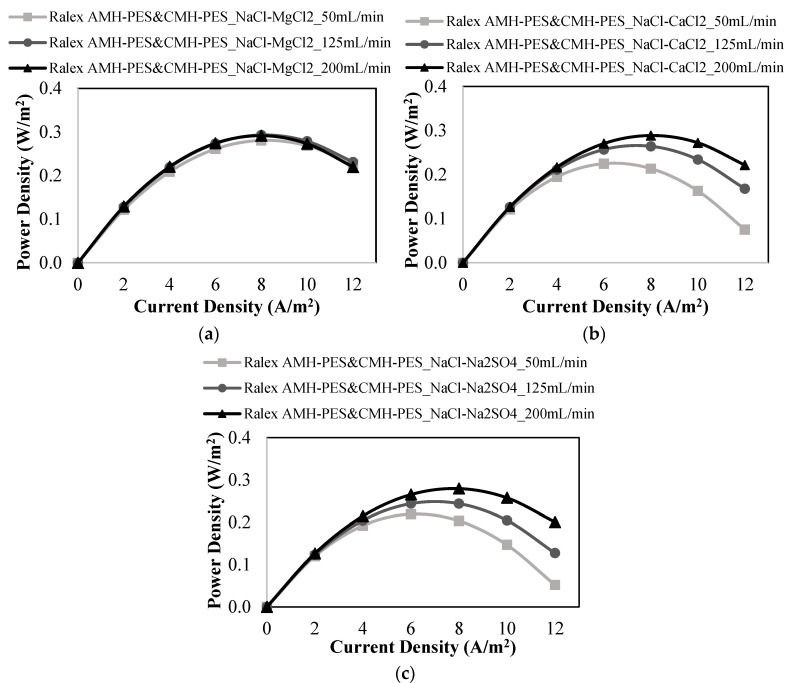
Power density vs. current density plots at different feed flow rates for the studies performed with (**a**) a mixture of NaCl and MgCl_2_, (**b**) a mixture of NaCl and CaCl_2_, (**c**) a mixture of NaCl and Na_2_SO_4_ using Ralex AMH-PES & CMH-PES membranes.

As can be seen from Figure 5, the power density results of studies performed with divalent ions (Mg^2+^, Ca^2+^, and SO_4_^2−^) were lower than that of the study performed with only NaCl.

In Figure 5a, the effect of Mg^2+^ ions on the resulting power density was obtained for three different feed flow velocities. As seen in Figure 5a, the power density results are very close to each other. The maximum power density was 0.291 W/m^2^ at the highest flow rate of 200 mL/min.

On the other hand, the power density results varied according to the flow rate in the presence of Ca^2+^ ions. As illustrated in Figure 5b, the power density increased with the increasing feed flow rate. The maximum power density obtained in the presence of Ca^2+^ ions was obtained as 0.289 W/m^2^ at 200 mL/min. The power density results of the studies performed in the presence of Mg^2+^ and Ca^2+^ ions were lower than that of the study performed with only NaCl (0.305 W/m^2^). So, the divalent cations (Mg^2+^ and Ca^2+^) have decreasing effect on the power generation due to their higher hydrated radii and lower diffusion coefficients than those of monovalent ions. The maximum power density in the presence of SO_4_^2−^ ions was 0.279 W/m^2^ at 200 mL/min (Figure 5c). This value was the lowest among the maximum power density results. Since divalent ions have higher valence than that monovalent ions, their affinities to the fixed groups of IEMs are stronger than those of monovalent ions [28]. Power density and OCV results of all studies performed with Ralex membranes are shown in Table 6. Thus, the divalent ions will be exposed to a higher membrane resistance than monovalent ions. In addition, divalent ions have a higher hydrated radius and lower diffusion coefficients than monovalent ions. So, divalent ions have low ionic mobility in the RED stack, and thus the ionic flux declined. Moreover, the transport of divalent ions through IEMs will be more difficult than those of monovalent ions.

For electroneutrality on the membrane sides, each divalent cation (Mg^2+^ or Ca^2+^) is exchanged with two monovalent cations (Na^+^) via the CEMs. This kind of transportation was originally known as uphill transport, which refers to the transfer of ions against a concentration gradient [19,28,29,30]. The multivalent ions triggered an increase in ohmic drop and raised the electrical resistance of the RED stack during the transport of Ca^2+^ or Mg^2+^ ions [28]. Furthermore, due to the low mobilities of divalent cations within the CEMs, the electrical resistances of CEMs increased, and a smaller electromotive force was observed by the addition of divalent cations (Mg^2+^ and Ca^2+^). The stack resistance will be higher and stronger in the existence of Mg^2+^ and Ca^2+^ ions according to Moreno et al. [30].

Other explanations for the observed phenomena include: (1) divalent counter ions have high valences, (2) divalent ions have high affinity to fixed functional groups of IEMs, (3) divalent ions have lower activity coefficients than monovalent ions, and (4) divalent ions have a larger hydrated radius than monovalent ions [28,31]. Depending on these explanations, OCV values obtained in the presence of divalent ions are low, and much lower power is generated by the RED system [18]. When OCV values obtained in our studies were examined, there was not any dramatic change in OCV values when monovalent and multivalent ions were added to feed solutions at various flow velocities. However, power density results obtained by the addition of multivalent ions (Mg^2+^, Ca^2+^, and SO_4_^2−^) were found to be lower than the values obtained with only NaCl salt, as expected.

Pintossi et al. [23] studied the influence of SO_4_^2−^ ions on the performance of power generation by a RED system and found that the presence of SO_4_^2−^ ions caused a decrease in permselectivity of membranes having high swelling degrees. This idea was supported by membrane selectivity measurements made in the mixture of NaCl and Na_2_SO_4_. For the studies performed with four different RED stacks, including five pairs of Neosepta CMX membranes and four different AEMs (Fujifilm type 1, Fujifilm type 10, Neosepta AMX, and monovalent ion selective Neosepta ACS membranes), it was concluded that increasing the fraction of SO_4_^2−^ ions in the feed solutions up to 50% (wt.) caused a decrease in power generation [23]. Rijnaarts et al. [31] and Moreno et al. [30] gave similar explanations for the influence of multivalent cations on CEMs. Because of the low permselectivity of membranes in the presence of multivalent ions, the electrical resistances of AEMs and CEMs have been observed as high [32]. The strong affinity of divalent ions by ion exchange membranes in the presence of divalent ions may lead to a significant increase in membrane resistance. The power generation performance of the RED system can be hampered depending on its high membrane resistance.

### 3.2. RED Studies Performed with Neosepta Membranes

In this case, the effect of monovalent (Na^+^, Li^+^, K^+^, and Cl^−^) and divalent ions (Mg^2+^, Ca^2+^, and SO_4_^2−^) were studied with five pairs of homogeneous Neosepta anion exchange (AMX) and cation exchange membranes (CMX).

**Figure 6:**
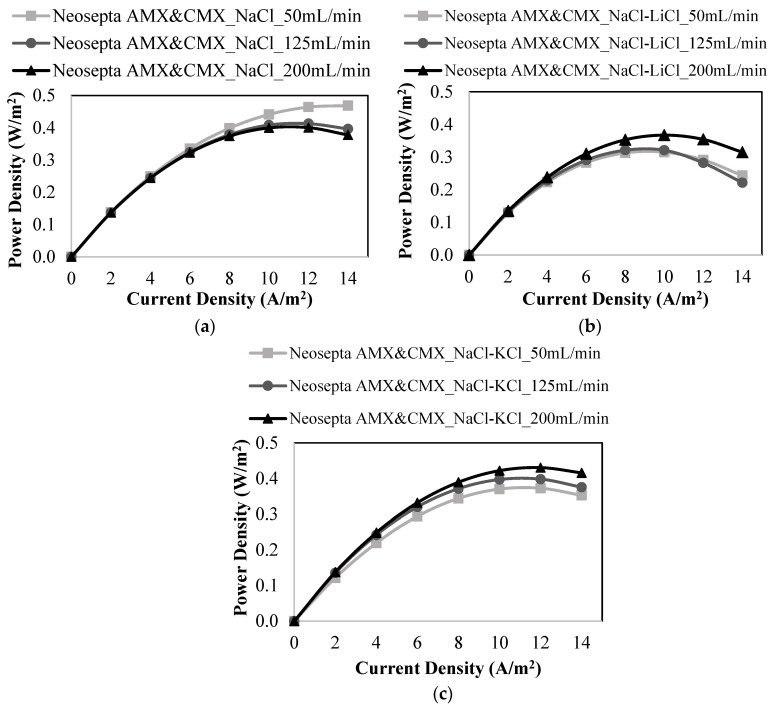
Power density vs. current density plots at different feed flow rates for the studies performed with (**a**) only NaCl, (**b**) a mixture of NaCl and LiCl, (**c**) a mixture of NaCl and KCl using Neosepta AMX & CMX membranes.

Again, the power generations achieved with different salt solutions were compared to the value obtained with feed solutions, including only NaCl. As seen in Figure 6a, with the feed solutions containing only NaCl, the generated maximum power density was 0.469 W/m^2^ at 50 mL/min. In the presence of LiCl and KCl salts along with NaCl, the respective maximum power density values were 0.367 W/m^2^ and 0.431 W/m^2^ at 200 mL/min. The power generation performance was badly affected by adding other monovalent ions, such as Li^+^ and K^+^ ions, to feed solutions having NaCl. Due to the higher hydrated radius and lower diffusion coefficients of Li^+^ ion than those of Na^+^ ion, Li^+^ ion has lower ionic mobility than Na^+^ ion. Thus, the ionic transportation of Li^+^ ions is restricted by CEMs due to the reasons explained above. Depending on the restrictions of CEMs to Li^+^ ions, the ionic flux slows down, and the power generation performance worsens.

Regarding the effect of the K^+^ ion, it has a larger atomic mass than that of the Na^+^ ion. Therefore, the addition of K^+^ ions into the feed solutions having NaCl will let the solution resistance increase. According to the literature, using high-resistance feed solutions reduces the power generation performance of the RED system [16].

The maximum power density values obtained by the Neosepta membranes were higher than those obtained by Ralex membranes due to the higher membrane thicknesses and membrane resistances of the Ralex membranes. Due to the heterogeneous characters of Ralex membranes, fixed charges are distributed non-uniformly in the structure of membranes. Consequently, the ion exchange mechanism across heterogeneous membranes is more complex. The performance of power generation using heterogeneous membranes is diminished in this case.

**Figure 7:**
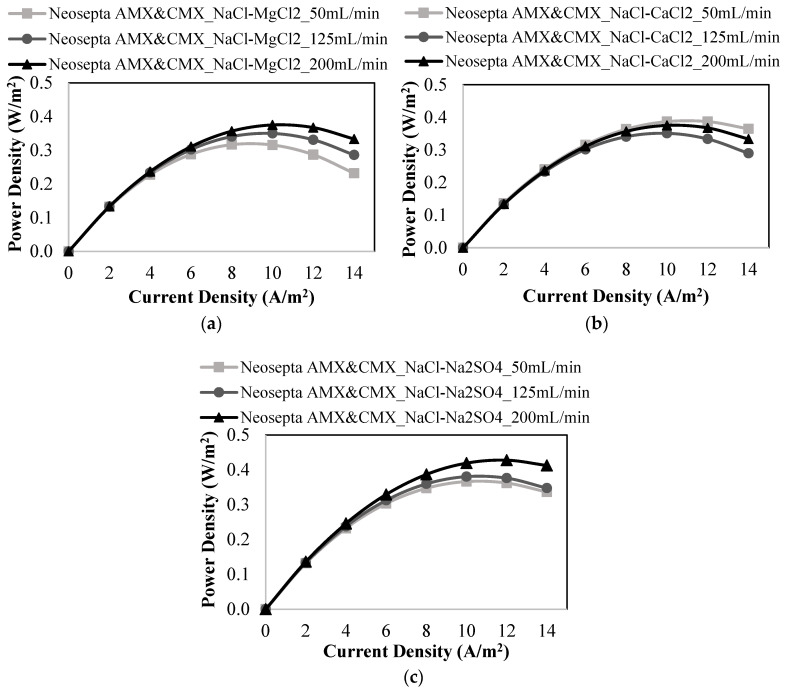
Power density vs. current density plots at different feed flow rates for the studies performed with (**a**) a mixture of NaCl and MgCl_2_, (**b**) a mixture of NaCl and CaCl_2_, (**c**) a mixture of NaCl and Na_2_SO_4_ using Neosepta AMX & CMX membranes.

Five pairs of Neosepta AMX and CMX membranes were used to evaluate the effect of divalent ions on power production in the RED system. For this, MgCl_2_, CaCl_2_, and Na_2_SO_4_ salts were sequentially coupled with 90% (wt.) of NaCl in feed solutions with a constant salt ratio of 1:30 (g:g). Figure 7 shows the effects of divalent cations (Mg^2+^ and Ca^2+^) and divalent anions (SO_4_^2−^) on the power density produced.

The maximum power density value obtained by employing a mixture of NaCl and MgCl_2_ in the feed solutions was 0.375 W/m^2^ at 200 mL/min, as shown in Figure 7a. The respective value for the mixture of NaCl and CaCl_2_ was 0.387 W/m^2^ at 50 mL/min (Figure 7b). According to the results obtained, it was considered that the presence of divalent cations caused some decrease in power generation for Neosepta membranes also. Figure 7c demonstrates the impact of SO_4_^2−^ ions on the power density. The maximum power density was 0.427 W/m^2^ at 200 mL/min. In the presence of SO_4_^2−^ ions in feed solutions, a lower power density was achieved compared to the study performed with only NaCl solutions. This situation results from higher valence, higher hydrated radius, and lower diffusion coefficient of SO_4_^2−^ ions than those of Cl^−^ ions (Table 1). Table 7 shows power density and OCV results of the studies carried out with Neosepta membranes.

When the maximum power densities obtained by Neosepta membranes were compared to those of Ralex membranes in studies performed in the presence of divalent ions, it was found that Neosepta membranes produced higher power density due to their lower membrane thickness, lower electrical resistance, and homogeneous structure.

### 3.3. RED Studies Performed with Fujifilm Membranes

Five pairs of homogeneous Fujifilm anion exchange (Fujifilm AEM Type II) and cation exchange (Fujifilm CEM Type II) membranes were employed to investigate the influence of coexisting ions (K^+^, Li^+^, Mg^2+^, Ca^2+^, and SO_4_^2−^).

**Figure 8:**
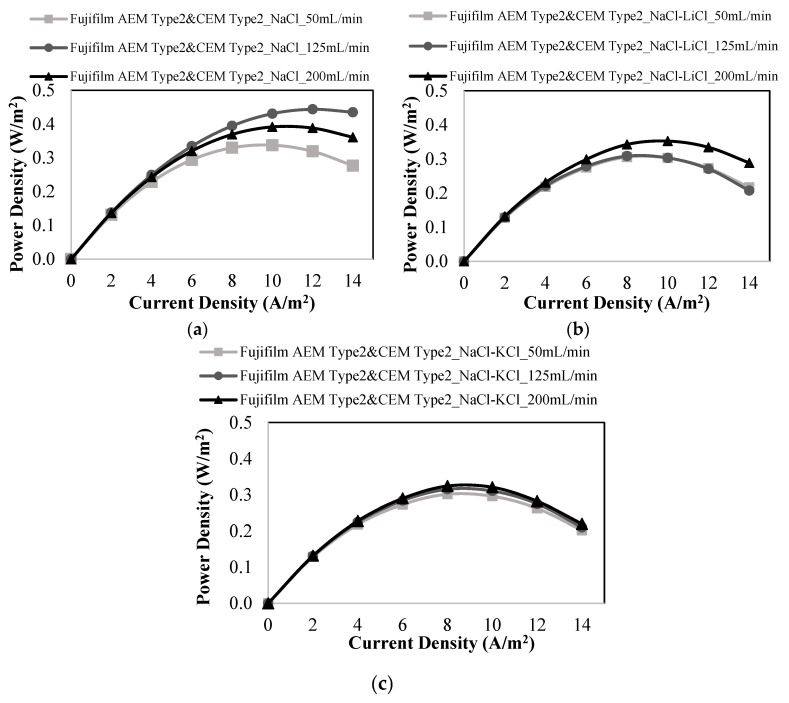
Power density vs. current density plots at different feed flow rates for the studies performed with (**a**) only NaCl, (**b**) a mixture of NaCl and LiCl, (**c**) a mixture of NaCl and KCl using Fujifilm AEM Type 2 & CEM Type 2 membranes.

The power density results of the studies with only Na^+^ ions were compared with those of the RED tests with salt mixtures containing Li^+^ and K^+^ ions. When only NaCl salt was employed, 0.444 W/m^2^ of maximum power density was attained at 125 mL/min of feed flow rates, as shown in Figure 8a. The maximum power density obtained by the addition of LiCl and KCl salts into NaCl solution was 0.353 W/m^2^ and 0.324 W/m^2^ at 200 mL/min, respectively (Figure 8b,c). The maximum power density values were obtained at different flow velocities in the studies performed with different salt mixtures. As shown in Figure 8, the addition of monovalent cations had a negative impact on power production by RED. Because the addition of monovalent ions caused the competition of ions during the passage through ion exchange membranes, and the studies performed with the addition of LiCl or KCl in the feed solutions resulted in a worse ion exchange process than that of the study performed with only NaCl. So, the power generation performance worsened in the presence of monovalent ions (K^+^ and Li^+^).

The results obtained by using different membrane pairs were compared to analyze the effect of monovalent ions which exist in the feed solutions on the power generation by RED. The studies performed using Fujifilm membranes gave the lowest power density values, whereas Neosepta membranes provided the highest power densities. Fujifilm membranes have lower ion exchange capacity than Ralex and Neosepta membranes. The reason for the higher power density of Ralex membranes than that of Fujifilm membranes may be the due to the higher ion exchange capacity of Ralex membranes providing enhanced ion transport.

**Figure 9:**
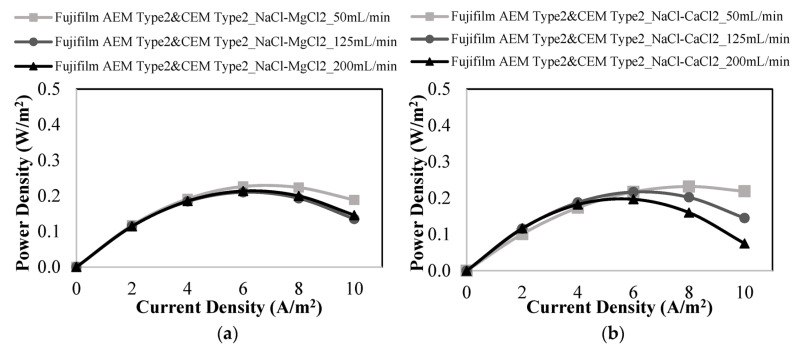

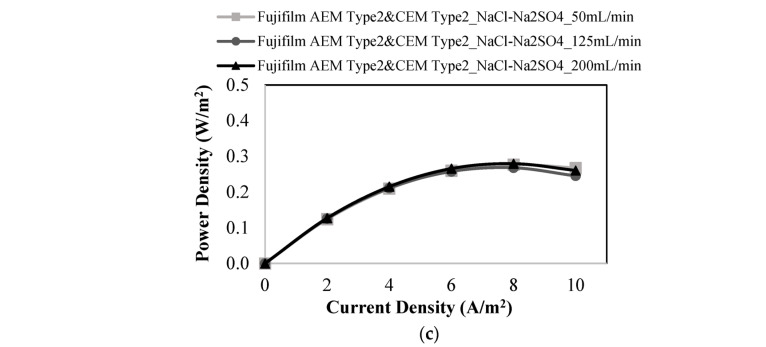
Power density vs. current density plots at different feed flow rates for the studies performed with (**a**) a mixture of NaCl and MgCl_2,_ (**b**) a mixture of NaCl and CaCl_2,_ (**c**) a mixture of NaCl and Na_2_SO_4_ using Fujifilm AEM Type 2 & CEM Type 2 membranes.

The effect of divalent ions on power generation by Fujifilm membranes was investigated using different binary salt combinations such as NaCl-MgCl_2_, NaCl-CaCl_2_, and NaCl-Na_2_SO_4_ (Figure 9). As demonstrated in Figure 9a,b, the presence of Mg^2+^ and Ca^2+^ ions lowered the performance of the power generation. At a linear flow rate of 50 mL/min, the maximum power densities were 0.232 and 0.226 W/m^2^ in the presence of Ca^2+^ ions and Mg^2+^ ions, respectively. The negative influence of Mg^2+^ ions on power generation was greater than that of Ca^2+^ ions because Mg^2+^ ions have a larger hydrated radius and lower diffusion coefficient than Ca^2+^ ions.

As shown in Figure 9c, when Na_2_SO_4_ was added to the feed solution, the power density decreased from 0.444 W/m^2^ produced when only NaCl was present in the feed solutions to 0.280 W/m^2^. Power density and OCV results of the studies performed with Fujifilm membranes are gathered in Table 8. The RED tests with Fujifilm membranes gave the lowest power densities when compared with the results of Ralex and Neosepta membranes. Neosepta membranes provided the highest power density values because of their homogeneous structure, low membrane thickness, and low membrane resistance. As stated before, the high mechanical strength of Ralex membranes may be caused to higher power generation performance than Fujifilm membranes.

## 4. Conclusions

In RED tests with feed solutions containing various ions, the greatest power density values were reached at various flow velocities. However, even at different flow rates, the OCV values were nearly identical. In none of the RED studies the effect of flow rate on power density and OCV was clearly observed. The addition of monovalent ions (K^+^ and Li^+^) had a variety of effects, depending on the ionic radius and diffusion coefficients of the ions, as well as the own properties of ion exchange membranes placed in the RED stack. In addition, monovalent ions are subjected to lower membrane resistance than divalent ions, allowing the RED system to generate more power. On the other hand, multivalent ions have a greater negative impact on open circuit voltage because of their higher charge and weaker permselectivity. The internal resistance of solutions containing divalent ions is higher than that of only NaCl, owing to lower diffusion coefficients and larger hydrated radius of Mg^2+^, Ca^2+^, and SO_4_^2−^ ions relative to Na^+^ and Cl^−^ ions. Thus, adding divalent ions to the feed solutions containing NaCl reduced the performance of power production in all trials. The addition of different salts to feed solutions containing NaCl had no discernible effect on the OCV results.

Neosepta AMX and CMX membranes were the least affected membranes in the presence of co-existing ions. It may be result of their higher ion exchange capacity and charge density. Neosepta membranes were the thinnest membranes among IEMs employed, and their electrical resistances and swelling degrees were lower than that of others. Nevertheless, broad characterization studies are needed to understand parameters affecting the transport of multivalent ions.

In contrast, power density and OCV values were examined for each IEM in the presence of coexisting ions, and Fujifilm AEM Type 2 and CEM Type 2 membranes were most affected by divalent ions, possibly due to their lower ion exchange capacity than other membranes. The performance of power generation with Neosepta membranes was higher than that of Ralex and Fujifilm membranes due to their higher charge density.

In future studies, for the enhancement of the power generation performance of the RED, studies performed with salt solutions having multivalent ions, membrane structures, and properties can be examined comprehensively.

## Figures and Tables

**Table 1:** Hydrated radius and diffusion coefficients of co-existing ions in feed solutions.

Ion	Hydrated Radius (×10^−10^ m) [20]	Diffusion Coefficient (×10^−9^ m^2^/s) [21,22]
Na^+^	3.58	1.334
Li^+^	3.82	1.029
K^+^	3.31	1.957
Ca^2+^	4.12	0.792
Mg^2+^	4.28	0.706
SO_4_^2−^	3.79	1.065
Cl^−^	3.32	2.032

**Table 2:** Operating parameters of the RED tests.

Parameter	Specification	
Effective area of membrane/electrode (cm^2^)	10 × 10	
Electrodes (anode and cathode)	Mesh type and alloyed with Ti/Ru-Ir (mesh 1.0, area: 10 × 10 cm)	
Thickness of spacer (µm)	465	
Volumetric flow rate of electrode solution (mL/min)	300	
Composition of electrode solution	Mixture of 0.05 M K_4_Fe(CN)_6_ and 0.05 M K_3_Fe(CN)_6_ in 0.25 M NaCl	
Salinity of feed solutions (g NaCl/L)	Low saline: 1	High saline: 30
Flow rates of feed solutions (mL/min)	50, 125, and 200	

**Table 3:** The properties of ion exchange membranes used in RED tests.

Specification	Ralex [15] AMH-PES	Ralex [15] CMH-PES	Neosepta [26] AMX	Neosepta [26] CMX	Fujifilm [27] AEM Type 2	Fujifilm [27] CEM Type 2
Type	Heterogeneous		Homogeneous			
Functionality	Anion exchange (Cl^−^ form)	Cation exchange (Na^+^ form)	Anion exchange (Cl^−^ form	Cation exchange (Na^+^ form)	Anion exchange (Cl^−^ form)	Cation exchange(Na^+^ form)
δ (μm)	714	700	140	170	210	190
IEC (mmol·g^−1^)	1.97	2.34	1.25	1.62	1.08 ± 0.05	1.35 ± 0.05
R (Ω·cm^2^)	7.66	11.33	2.0–3.5	2.0–3.5	5	8
α (%)	89.3	94.7	91.0 ± 0.4	92.5 ± 0.6	95	96
SD (%)	56.0	31.0	16.4 ± 0.5	21.5 ± 0.2	-	-
CD (meq·g^−1^ H_2_O)	3.5	7.6	5.4	9	-	-
BS (kg.cm^−2^)	-	-	4.5–5.5	3.5–6.0	5.0	4.7

δ: membrane thickness, IEC: Ion exchange capacity, R: electrical resistance, α: permselectivity, SD: Swelling degree, CD: Charge density, BS: Burst strength. Electrical resistance: Equilibrated with a 0.5 M NaCl solution at 25 °C.

**Table 4:** Quantities of salt used in feed solutions.

Salt Pairs Used in Feed Solutions		LCC ^1^ (M)	HCC ^2^ (M)
NaCl		0.0171	0.5128
NaCl-LiCl	NaCl	0.0154	0.4615
	LiCl	0.0024	0.0708
NaCl-KCl	NaCl	0.0154	0.4615
	KCl	0.0013	0.0402
NaCl-CaCl_2_	NaCl	0.0154	0.4615
	CaCl_2_	0.0009	0.0270
NaCl-MgCl_2_	NaCl	0.0154	0.4615
	MgCl_2_	0.0011	0.0315
NaCl-Na_2_SO_4_	NaCl	0.0154	0.4615
	Na_2_SO_4_	0.0007	0.0211

^1^ Low concentrated compartment. ^2^ High concentrated compartment.

**Table 5:** Types of salt mixtures used for different membrane pairs.

Membranes	Binary Salt Mixtures Used in Feed Solutions
Ralex AMH-PES & CMH-PES	NaCl
	NaCl + LiCl
Neosepta AMX & CMX	NaCl + KCl
	NaCl + MgCl_2_
Fujifilm AEM Type 2 & CEM Type 2	NaCl + CaCl_2_
	NaCl + Na_2_SO_4_

**Table 6:** Power density and OCV results of the studies performed by Ralex AMH-PES and Ralex CMH-PES membranes.

Membranes	Flow Rates of Feed Solutions (mL/min)	Salt Mixtures in Feed Solutions	Power Density (W/m^2^)	OCV (V)
Ralex AMH-PES & Ralex CMH-PES	50	NaCl	0.284	0.742
	125		0.289	0.743
	200		0.305	0.749
	50	NaCl-LiCl	0.226	0.727
	125		0.267	0.731
	200		0.306	0.736
	50	NaCl-KCl	0.296	0.722
	125		0.309	0.742
	200		0.330	0.743
	50	NaCl-MgCl_2_	0.243	0.694
	125		0.242	0.733
	200		0.291	0.732
	50	NaCl-CaCl_2_	0.225	0.717
	125		0.264	0.733
	200		0.289	0.726
	50	NaCl-Na_2_SO_4_	0.219	0.718
	125		0.244	0.723
	200		0.279	0.728

**Table 7:** Power density and OCV results of the studies performed by Neosepta AMX and Neosepta CMX membranes.

Membranes	Flow Rates of Feed Solutions (mL/min)	Salt Mixtures in Feed Solutions	Power Density (W/m^2^)	OCV (V)
Neosepta AMX & Neosepta CMX	50	NaCl	0.469	0.755
	125		0.413	0.756
	200		0.400	0.759
	50	NaCl-LiCl	0.315	0.735
	125		0.321	0.746
	200		0.367	0.753
	50	NaCl-KCl	0.373	0.666
	125		0.398	0.752
	200		0.431	0.759
	50	NaCl-MgCl_2_	0.317	0.744
	125		0.350	0.750
	200		0.375	0.743
	50	NaCl-CaCl_2_	0.387	0.757
	125		0.351	0.748
	200		0.375	0.743
	50	NaCl-Na_2_SO_4_	0.366	0.738
	125		0.380	0.748
	200		0.427	0.752

**Table 8:** Power density and OCV results of the studies performed by Fujifilm AEM Type 2 and CEM Type 2 membranes.

Membranes	Flow Rates of Feed Solutions (mL/min)	Salt Mixtures in Feed Solutions	Power Density (W/m^2^)	OCV (V)
Fujifilm AEM Type 2 & Fujifilm CEM Type 2	50	NaCl	0.338	0.735
	125		0.444	0.757
	200		0.392	0.756
	50	NaCl-LiCl	0.306	0.732
	125		0.309	0.738
	200		0.353	0.741
	50	NaCl-KCl	0.302	0.743
	125		0.315	0.749
	200		0.324	0.750
	50	NaCl-MgCl_2_	0.226	0.692
	125		0.210	0.685
	200		0.213	0.677
	50	NaCl-CaCl_2_	0.232	0.572
	125		0.217	0.608
	200		0.197	0.705
	50	NaCl-Na_2_SO_4_	0.277	0.714
	125		0.268	0.731
	200		0.280	0.733

## Data Availability

The data available in this study are available on request from the corresponding author.
